# Mobile Phone-Based Nutrition Education Targeting Pregnant and Nursing Mothers in Sri Lanka

**DOI:** 10.3390/ijerph20032324

**Published:** 2023-01-28

**Authors:** Dilka Rashmi Peiris, Millawage Supun Dilara Wijesinghe, Balangoda Muhamdiramlage Indika Gunawardana, Weerasinghe Mudiyanselage Prasad Chathuranga Weerasinghe, Rajapaksha Mudiyanselage Nayani Umesha Rajapaksha, Kumari M. Rathnayake, Nayomi Ranathunga, Saman Kalupahana, Yakupitiyage Asanka Supun, Sameer Deshpande, Faruk Ahmed

**Affiliations:** 1 Scaling Up Nutrition People’s Forum, Colombo 00700, Sri Lanka; 2 Health Promotion Bureau, Colombo 01000, Sri Lanka; 3 Department of Applied Nutrition, Faculty of Livestock, Fisheries and Nutrition, Wayamba University of Sri Lanka, Makandura 60170, Sri Lanka; 4 Department of Physiology, Faculty of Medicine, Wayamba University of Sri Lanka, Kuliyapitiya 60200, Sri Lanka; 5 World Food Program, Colombo 00500, Sri Lanka; 6 Social Marketing @ Griffith, Griffith University, Nathan 4111, Australia; 7 Public Health, School of Medicine and Dentistry, Griffith University, Gold Coast 4222, Australia

**Keywords:** mobile intervention, mHealth, knowledge on nutrition, attitudes on nutrition, dietary practices, nutrition in lactating period, nutrition in pregnancy

## Abstract

Introduction: A woman’s nutrition during pregnancy and nursing affects the mother and the growing child. Similarly, the first two years of a child’s life are critical to their growth and development and are facilitated by optimum nutrition. Women’s nutrition-related knowledge, attitudes, and practices influence household food and nutrition security. Mobile health (mHealth) is a potentially effective health intervention in pandemic situations when physical gatherings are restricted. Objectives: To examine the effectiveness of a mobile phone-based nutrition education intervention targeting pregnant and nursing mothers in six Sri Lankan divisional secretariat areas. Method: This intervention was evaluated using a before and after within-subjects design. The intervention included 19 messages over four weeks sent via mobile phone, covering nutrition themes such as pregnancy care, infant and young child-feeding, diet, family care for mother and child, and cash management. The intervention was evaluated based on a quantitative survey using a structured interviewer-administered questionnaire and qualitative interviews using a semi-structured questionnaire. The study population was pregnant and nursing mothers. The objective of the qualitative interviews was to identify how respondents used messages and how satisfied they were with the project. The outcome measures were awareness/knowledge, attitudes, social norms, self-efficacy, behaviour intentions, and practices of pregnant and nursing mothers. Trained enumerators collected data using a mobile phone. Results: A total of 996 pregnant and nursing mothers participated in the pre-assessment survey, of which 720 completed the post-assessment. Most were nursing mothers (84.2% pre- and 78.9% post-assessment). Participants provided positive feedback on the intervention. Knowledge/awareness (t = −18.70, *p* < 0.01) and attitudes (t = −2.00, *p* < 0.05) increased when exposed to the intervention. Favourable improvements in the practices were also observed. Mothers’ practices related to breastfeeding and 24-h dietary diversity showed a statistically significant improvement. However, social norms and behaviour intentions did not significantly improve. The qualitative component also revealed favourable responses. Conclusion and Recommendations: The mobile intervention improved participants’ knowledge, awareness, attitude, and practices, but not social norms or behaviour intentions. This approach is recommended to be used on a larger scale in community settings. In addition, mobile technology could drive intervention in pandemic-related situations.

## 1. Background

### 1.1. Situation and the Need for Nutrition Message Sharing

A woman’s nutritional status during pregnancy and breastfeeding is critical not only for her health but also for her future generations. Pregnancy and lactation periods are two crucial stages that need sufficient nutrition in the life cycle. During these periods, there is an increased physiological demand for nutrients for foetal growth, milk production, and the baby’s healthy development. The nutritional needs of women increase during pregnancy and breastfeeding to support all of these changes, prepare the body for delivery and breastfeeding, and ensure the normal development of the foetus/baby. Thus, a personalised approach to nutritional advice is recommended [1]. Therefore, maternal nutrition, knowledge, and practices are of utmost importance for the mother’s well-being, the foetus’ development, and the child’s growth.

As another example, proper growth and development are facilitated by the optimum nutrition of the child. This becomes important in the first two years of a child’s life. Exclusive breastfeeding is encouraged in the first six months, and complimentary food later. All related healthcare staff are encouraged to empower the family members on properly adhering to “Infant and Young Child Feeding” guidelines [2].

In Sri Lanka, among children aged below five, 17% are stunted, 21% are underweight, and 15% are wasted. The low birth-weight rate of children is 15.7% [3]. Micronutrient deficiency conditions, especially anaemia, are also a high burden among pregnant women and children, causing the country to bear a triple burden of malnutrition [4]. In recent years, the prevalence of women with a body mass index of less than 18.5 kg/m^2^ was 22.5%, which was documented to decline with increasing educational levels [5]. The prevalence of anaemia among pregnant women was known to be 31.8%. A significant decline in anaemia was seen with increasing levels of education among women [5].

### 1.2. Need for Nutrition Education through a Personalised Approach Using Mobile Phones

Nutrition education aims to reinforce specific nutrition-related practices or behaviours to change habits that contribute to poor health; this is done by motivating change among people to establish desirable food and nutrition behaviour to promote and protect good health. In addition, people are given help to learn new nutrition information and develop the attitudes, skills, and confidence needed to improve their nutrition practices [6]. A woman’s nutritional status and knowledge are potent indicators of her children’s nutritional status and household food security. Accordingly, food and nutrition-related knowledge, attitude, and practice (KAP) are critical to achieving healthier dietary practices, household food, and nutritional security [7].

Sri Lanka has a free and well-established primary health care service delivery system. The public health midwife (PHM) is the grassroots link between government health services and mothers concerning maternal and child health (MCH) services. Midwives provide domiciliary care and advice to mothers and are responsible for an ideal population of 3000. However, many health units exceed this limit, resulting in a heavy burden of work and less time to spend with every mother. Therefore, using mobile health in a setting with limited resources is beneficial for a developing country like Sri Lanka. Previously, Sri Lanka has not systematically used expert-led nutrition messages with mobile phones in the primary health care setting.

Mobile phones are extensively used by the population and are widely adopted for health interventions to improve health conditions. Compared to traditional methods, with lower cost, feasibility, and the number of mobile phone users, the ability to engage and impact many people has made mobile phone-based health messages a crowd-pleasing and popular form of health intervention [8].

mHealth as a tool for health interventions is widely used worldwide. It has emerged as a promising tool to address access, coverage, and equity gaps in developing countries and low-resource settings [9]. Success stories of mHealth have been documented in Asia. A systematic literature review conducted in Sub-Saharan Africa and southern Asia mentioned that simple mHealth educational interventions based on SMS and voice message reminders effectively supported behaviour changes in pregnant women and training of health workers, thus improving antenatal and postnatal care attendance, vaccination coverage, and skilled birth attendance [10]. Mobile platform-based interventions remain a promising avenue for education and information dissemination, particularly for health-related topics that carry a significant potential to improve individual and population health and well-being, such as maternal and reproductive health. Pregnancy and postpartum care have been targeted due to the high risks to mothers and infants during this period [11].

According to the Telecommunications Regulatory Commission of Sri Lanka (TRCSL) statistics, the number of mobile phone subscriptions by June 2022 was 29.56 million, and the cellular mobile voice telephony density (per 100 inhabitants, as of December 2021) was 135. According to a 2019 survey, only 47% of all mobile phone users owned smartphones [12], and internet penetration in Sri Lanka stood at 50.8% in January 2021 [13].

Due to the COVID-19 pandemic in 2021, healthcare staff faced an immense difficulty in providing services. The health and nutrition service providers regularly delivered health and nutrition awareness and education programs amidst difficulties. They faced challenges while providing healthcare services amidst the pandemic, with fewer human resources, and physical and distance barriers leading to difficulties in hosting antenatal clinics, delivering sessions regularly, and growing caseloads causing an intense burden on the health sector [14,15]. This necessitated identifying alternative means of delivering nutrition-related messages. Mobile phones offered an effective alternative to ensure the continuity of the programs.

### 1.3. Previous Studies

There is worldwide evidence from interventions using mobile phones to deliver messages targeting various audiences. We have highlighted a few examples. The Mobile Alliance for Maternal Action (MAMA) supports country programs in Bangladesh, India, South Africa, and Nigeria. It delivers vital health messages to new and expectant mothers via their mobile phones’ mHealth SMS text messaging service to improve maternal, foetal, and infant health outcomes [16].

“Aponjon” is a mobile phone-based mHealth service for expecting and new mothers in Bangladesh, operated under the MAMA program [17]. The Aponjon service offers information for pregnant women and new mothers via mobile phone voice or text messages. This intervention targets to achieve health-related outcomes focused on pregnancy, maternal and child health services, birth, postpartum, or newborn care, and does not target the nutrition-related messages of improving maternal dietary intake. A woman can enrol in the service at any time during her pregnancy and continue to receive phone messages until the baby turns one year of age. Chowdhury et al. found that using Aponjon services for at least six months during pregnancy was associated with increased knowledge and positive behaviours, and knowledge of newborn healthcare after delivery [18].

An intervention in Uganda aimed to study the experiences of village health teams who used nutrition and health-related educational videos displayed on mobile phones during their family visits, and to analyse mothers’ reflections on this video education [19]. Community health workers (CHWs) and mothers considered that the videos had more strongly impacted their learning than traditional teaching methods; they felt the videos improved the child-feeding and caring competence of both CHWs and mothers.

In Bangladesh, another mHealth project successfully linked village doctors with trained doctors [20]. Additionally, decade-long stories of Asia’s mHealth innovations were documented, especially on grassroots challenges and practical interventions [21].

Alam and others [22] have also documented that low-cost mobile phone educational services work as catalysts in improving maternal and child health behaviour in resource-limited settings. The results indicate that mobile phone texts or voice messages may delay the timing of the first bath within the first 48 h after birth. This type of mobile phone voice or text message-sharing offers information on pregnancy, delivery, essential newborn care, and nutrition for pregnant women and new mothers.

Several reviews have stated that mHealth initiatives are being increasingly used and tested to improve healthcare delivery in low- and middle-income countries [23]. Previous studies, reviews, and meta-analyses have shown an increase in antenatal care (ANC) visits and delivery by skilled birth attendants among women who received text message reminders about ANC appointments and health information during their pregnancy and newborn care [24].

A qualitative study carried out in hilly areas in Nepal suggested that text message-based mHealth interventions in under-resourced settings could be encouraged and may result in higher service coverage and ultimately improved health and nutrition practices [25].

The abovementioned interventions did not measure effectiveness, but the following intervention reported effectiveness. In Gujarat, India, the use of mobile- and web-based applications as a job aid by government accredited social health activists (ASHA) and primary healthcare staff improved the coverage and quality of maternal nutrition and child health services in hard-to-reach areas [26].

## 2. Review of mHealth Interventions in Sri Lanka

No published studies on interventions were found in Sri Lanka relevant to sharing mobile phone-based messages using text, social media, or app-based messages targeting pregnant women and nursing mothers. In Sri Lanka, a qualitative study by Weerasinghe [27] recommends that the mHealth platform could be a promising initiative to strengthen the existing face-to-face nutritional advice the field health workers provide to improve the nutritional status of children. The study was limited to the tea estates worker communities and studied the nature of mobile phones used in this community and their perceptions on using mHealth counselling for infant and young child feeding. Our intervention fills the gap and is the first documented effort that used mHealth (both text and social media) to deliver messages in Sri Lanka. The COVID-19 pandemic also created an environment where people have limited physical access to health services. Therefore, it accentuated the need for mHealth technology.

To summarise, this study examines the effectiveness of a mobile phone-based nutrition education intervention targeting pregnant and nursing mothers in six Sri Lankan divisional secretariat areas. This intervention was evaluated using a before and after within-subjects design, described in detail next.

## 3. Methods

### 3.1. Study Design, Setting and Participants

The intervention was evaluated with two waves of data collection. This included a quantitative and qualitative assessment conducted in pre- and post-intervention periods. Participants were from selected divisional secretariat areas belonging to six districts of Sri Lanka: Batticaloa, Mannar, Matale, Monaragala, Mullaitivu, and Kalutara. Pregnant and nursing mothers whose families were in the low-income category were eligible to participate in the study. A total of 1350 participants were invited to participate in the intervention through a phone call. Pregnant and nursing women who had been residing in the areas for the last six months and agreed to receive the phone calls and messages of the intervention were included. Pregnant women and nursing mothers who had significant illnesses or difficulties in participation due to current COVID-19 situations, or who have come temporarily to stay in the area, were excluded from the study.

The hierarchy of effects model (HOEM) was used to evaluate the intervention [28,29]. The conceptual model used in the evaluation is shown in Table 1.

### 3.2. Ethics Approval

Ethical clearance was obtained from the Ethics Review Committee of two universities: Griffith University, Australia, and the Faculty of Medicine, Wayamba University of Sri Lanka. Verbal and short mobile phone text-based consent was obtained from the participants.

### 3.3. The Intervention

The intervention was designed and implemented by the Scaling Up Nutrition People’s Forum, in collaboration with the Family Health, Nutrition Communication & Behaviour Research Unit of the Health Promotion Bureau of the Ministry of Health, Sri Lanka, and Griffith University, Australia; the project was funded by World Food Program, Sri Lanka. The campaign theme was “Poshanayayi Piripun Pawlayi” (in Sinhala) and “Arivulla kudumpamum-Aarookkiyamaana kulanthaiyum” (in Tamil), which highlights the importance of good nutrition and the fullness of family life.

**Figure 1:**
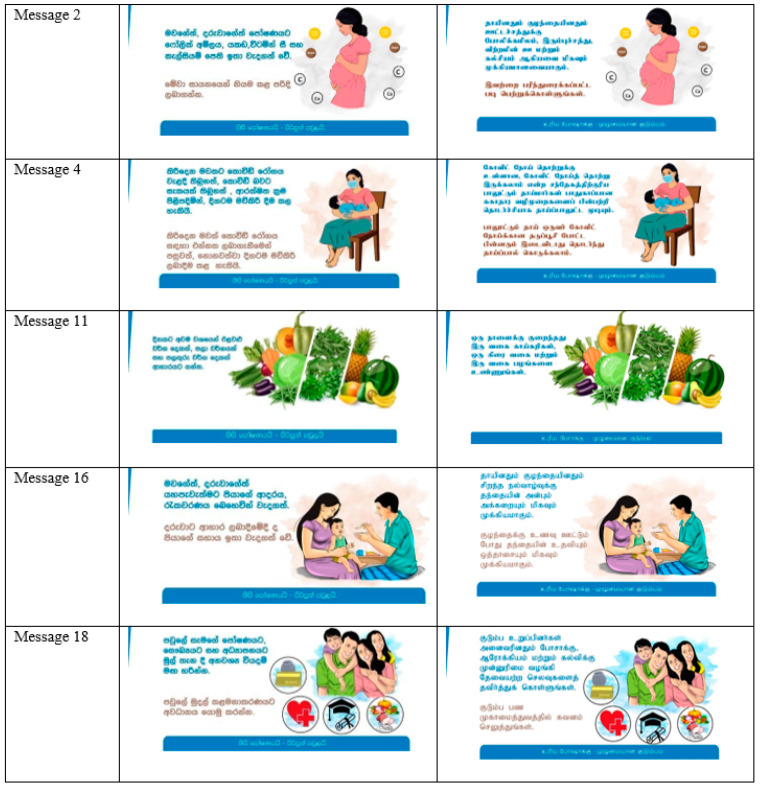
Examples of the social media messages shared. Refer to Table 2 for English translation.

The intervention delivered 19 messages through mobile phones in November 2021, such as text messages (bulk short messaging system) and social media (i.e., WhatsApp, Viber, YouTube videos) (Figure 1). These nutrition-related messages were developed by a team consisting of nutritionists, consultant community physicians, health education officers, members of the Nutrition Society of Sri Lanka, and active members of NGOs involved in nutrition-related activities at the ground level. Messages were formulated to address identified problem areas based on the available evidence and the experiences of the team of experts based on standard health communication principles. The target group of pregnant and nursing mothers belonged to two main ethnic groups, Sinhalese and Tamil, in six Sri Lankan districts; the messages were shared using both languages. These messages covered five nutrition-related domains, as mentioned in Table 2. The messages were pre-tested with a similar group of 30 mothers to check whether they were easily readable, informative, understandable, influential, related, attractive, and motivating to take a call to action.

First, the team shared short text messages with all participants, two daily messages for ten days. Later, two social media messages were shared every day for ten days.

Follow-up calls were made to every participant after the team had delivered 8–10 messages. The intention was to ask whether they had received and read the messages. They were thanked if they had read the messages or were reminded to read them. See Figure 1 for examples of the pictures of the social media messages shared in local languages.

### 3.4. Study Tool and Data Collection

In both assessments, study tools included a structured interviewer-administered questionnaire using mobile phones. The quantitative questionnaire included socio-demographic characteristics, phone literacy, and preferences for mass media. Pre-assessment and post-assessment consisted of questions relevant to awareness and knowledge, attitudes, social norms, self-efficacy, intentions, and practices, as described in Table 1. Some questions were adopted from already published studies [29], and we modified the questions to match our intervention. A team comprising content experts in nutrition, behaviour change, and health communication validated the content of the questionnaire. The questionnaire was prepared for data collection using the Kobo Toolbox, and in-built error checking was carried out. The questionnaire was pre-tested over the phone with 33 target participants. Pre-testing helped to identify the gaps and finalise the questionnaire.

Awareness and knowledge were assessed using a multi-item questionnaire consisting of 15 questions. For example, “what nutrition supplements does the clinic provide during pregnancy?” There were multiple answers to this question, and the responses were categorized into two to make it dichotomous. Likert scales (1–5) were used to measure attitudes (five questions), social norms (five questions), self-efficacy (five questions), and intentions (three questions), as reflected in Table 1.

The pregnant women and nursing mothers’ minimum dietary diversity of women (MDD-W) was calculated [30]. MDD-W is a dichotomous indicator of whether women 15–49 years of age have consumed at least five of ten defined food groups the previous day or night. The proportion of women 15–49 years of age who reach this minimum in a population can be used as a proxy indicator for higher micronutrient adequacy, one important dimension of diet quality.

A qualitative assessment was conducted using 30 interviewees over the phone with a semi-structured interviewer-administrated questionnaire. Some of the questions asked were: Did you receive messages on your phone during the past month? Did you read them? How helpful were the messages, and how did you employ the recommended behaviours? Did you share them with anyone, and with whom?

### 3.5. Data and Statistical Analysis

Pre-post-matched analysis informed the influence of the intervention. Names and phone numbers were matched for the pre-and post-assessments, and the participants in the pre-assessment were cross-referenced by their birth year and date. All identifying information was removed from the data to comply with ethics. Before the primary analysis, data on knowledge/awareness, attitudes, social norms, self-efficacy, behavioural intentions, and practices were checked for normality. Data were analysed using Statistical Package for Social Sciences (SPSS) version 24. Descriptive analyses were conducted with frequency distribution for nominal and means and standard deviations for continuous variables. The matched pre-post means for continuous variables were analysed using paired t-tests. Categorical variables were analysed using the Chi-squared test. The statistical significance value was taken as *p* < 0.05.

## 4. Results

### 4.1. Quantitative Assessment Findings

**Figure 2:**
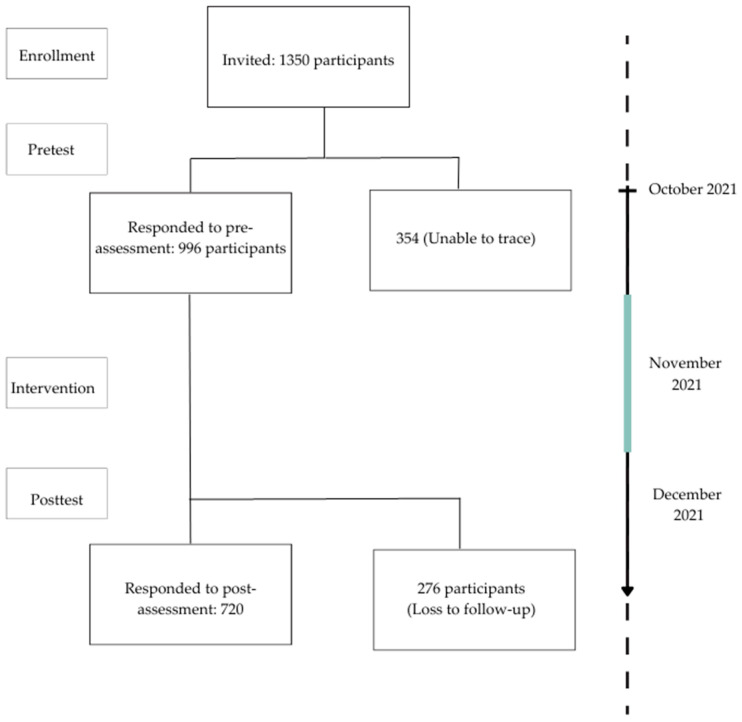
Flow diagram of the study participants in each stage.

Of the 1350 invited individuals, 996 responded in the pre-assessment, with a response rate of 73.7%. The team was unable to trace 354 participants due to incorrect contact details. In the post-assessment, 720 participants responded, with a response rate of 72.3% (720/996) (Figure 2).

Basic socio-demographic characteristics (district, age, respondent status, language reading, level of education, ownership of mobile phone, usage of TV/radio) of the participants who were lost to follow-up did not statistically differ from the study participants.

#### 4.1.1. Socio-Demographic Characteristics

In the study sample, most of the study participants were nursing mothers (84.2%, *n* = 839). The mean (SD) age of the study participants was 28 (5.56) years, and the majority (59.6%, *n* = 594) were 21–30 years old. Approximately half of the participants used Tamil as their language of reading (51.8%, *n* = 516) (Table 3).

Most participants acquired nutrition and gender-related messages from PHMs (95.8%, *n* = 954). The mean (SD) age of the first child was 4.5 (3.33) years, and the mean birth weight of the first child was 2916.63 g (SD = 473.77). More than two-thirds of the participants owned a personal mobile phone (67.6%, *n* = 673), while the majority had feature phones (56%, *n* = 558) (Table 3).

#### 4.1.2. Awareness/Knowledge, Attitudes, Social Norms, Self-Efficacy, and Behaviour Intentions of the Sample

In the post-assessment, the knowledge, attitude, social norms, and behaviour intentions of the sample were higher than in the pre-assessment. However, only knowledge (t = −18.70, *p* < 0.01) and attitude (t = −2.00, *p* < 0.05) showed a statistically significant difference (Table 4).

Furthermore, a separate analysis was carried out among pregnant women (*n* = 120). It was shown that the level of knowledge related to nutrition during pregnancy significantly increased (pre mean (SD) = 4.45 (1.25), post mean (SD) = 5.01 (1.06), t = −3.10, *p* < 0.01), while attitudes (pre mean (SD) = 15.16 (1.88), post mean (SD) = 15.17 (1.65), social norms (pre mean (SD) = 7.74 (1.52), post mean (SD) = 7.64 (1.10), and behaviour intentions (pre mean (SD) = 10.55 (1.23), post mean (SD) = 10.54 (1.20) were not statistically significant.

Similarly, a separate analysis was conducted only for the nursing mothers (*n* = 600). It was shown that the level of knowledge (pre mean (SD) = 3.82 (1.10), post mean (SD) = 4.85(1.11), t = −16.85, *p* < 0.01), and the self-efficacy (pre mean (SD) = 4.26 (0.86), post mean (SD) = 4.37 (0.97), t = −1.98, *p* < 0.05), significantly increased, while attitudes (pre mean (SD) = 15.23 (2.42), post mean (SD) = 15.38 (2.35), social norms (pre mean (SD) = 7.92 (1.45), post mean (SD) = 7.88 (1.65), and behaviour intentions (pre mean (SD) = 5.15 (0.76), post mean (SD) = 5.21 (0.81) were not statistically significant.

A separate analysis was conducted based on phone ownership and the type of phone. The overall knowledge, attitudes, social norms, and behaviour intention of the sample who owned any mobile phone were positive. However, only the level of knowledge (t = −15.56, *p* < 0.01) and the attitude (t = −2.98, *p* < 0.01) showed statistical significance (Table 5).

Similarly, we analysed those who did not own a personal mobile phone (but used spouse or family members’ phones). Only the level of knowledge (t = −10.28, *p* < 0.01) showed a statistically significant difference (Table 5).

Based on the type of phone (smart/feature), the sample was again re-analysed. Only the level of knowledge showed a statistically significant difference between pre- versus post-assessments among smartphone users (t = −15.06, *p* < 0.01) and feature phone users (t = −11.60, *p* < 0.01).

#### 4.1.3. Practices of the Sample

Practices related to breastfeeding and the 24-h dietary diversity based on consumption of 10 main food groups using the minimum dietary diversity for women indicator (MDD-W) were assessed. All practices showed a statistically significant improvement (Table 6).

#### 4.1.4. Overall Campaign Feedback

Overall campaign feedback was assessed using a 5-point Likert scale (very useful to very useless); 59.9% (*n* = 431/720) believed the project was very useful, while 38.8% (*n* = 279/720) found it helpful.

### 4.2. Qualitative Findings

Thirty beneficiaries participated in the pre- and post-qualitative assessments. Out of the 30 who participated in the post-assessment, 17 had participated in the pre-assessment. Interviews were conducted on the phone in Sinhala and Tamil and back-translated to English.

The following participant quotes reflect the intervention effect:

- *“I changed the diet patterns of my family. I added two vegetables and one green leafy vegetable. I eat two fruits as many days as possible. Also, I breastfeed on demand; previously, I did it once in two hours.”*
- *“We have changed our cash management patterns. Now we are more focused on buying nutritious food items, vegetables, and fruits, and we have also changed the food-intake patterns.”*
- *“Earlier, I breastfed the baby once in 2 h. Now I take care of my child more and feed the baby when she needs it. This is the most important thing that I changed after receiving these messages. Moreover, we have increased the vegetables and fruit intake now.”*
- *“After receiving these messages, I have been influenced to think about how to save money and what essential things should be bought. Usually, I am very concerned about nutritious foods. Although I had restricted to low sugar intake, I did not practice it for salt. After receiving these messages, I now avoid adding salt to the rice and have a restricted salt intake. It would be good for our health.”*

The feedback was received on sharing messages with other family members and receiving support from them. After sharing the messages with husbands and family, 18 beneficiaries received support from husbands and family members for child feeding, child caring, and household activities. According to eight participants, family members, including the husband, supported adopting new dietary patterns (e.g., reducing salt and adding green leafy vegetables for meal preparation). Twenty-two beneficiaries highlighted that they learned new/essential things from those messages, while one beneficiary was influenced to change. In addition, one participant’s husband discussed a plan to change their expenditure pattern with her. Another husband supported the beneficiary in maintaining the nutrition level of the family. A nursing mother had planned on giving complementary feeding to her baby after getting messages from one participant.

- *“My husband also read all messages. Now, he supports me in maintaining the nutritional status of my family.”*
- *“As I shared these messages with my husband, especially gender-based messages, his support for me to do household activities and child-caring has significantly increased. It was very beneficial to me.”*

Most beneficiaries requested to continue this project with more details. Most of them said this is a worthwhile project amidst the COVID-19 situation. A few requested that this project was delivered for all, not just selected mothers.

- *“If you can send these kinds of messages continuously for us, it would be beneficial. Also, if you can add more details, it would be more effective as providing more details would influence us more to adopt new behaviours.”*
- *“This was a good program. We received valuable messages from you. Nowadays, we cannot go to the clinic to attend awareness sessions. As you sent messages via phone, we could receive messages while in the home.”*
- *“This project was beneficial, and the messages shared with us were very informative. I am expecting to get more messages from you. Now my baby is six months old. From next month I have to start complementary feeding. I need more information regarding complementary feedings, such as food varieties, preparation methods, amount, and texture. Continuously send these messages. We are happy to receive and ready to change our behaviours.*
- *“Due to this COVID situation, I know it is difficult to conduct face-to-face programs. Therefore, expand this project to other areas.”*

## 5. Discussion

This is the first documented large-scale mobile-based nutritional intervention targeting pregnant and nursing mothers in six Sri Lankan districts. Post-assessment revealed that the knowledge/awareness (t = −18.70, *p* < 0.01) and attitudes (t = −2.00, *p* < 0.05) increased when exposed to the intervention. Although social norms and behaviour intentions increased, they did not significantly improve. Mothers’ practices related to breastfeeding and 24-h dietary diversity showed a statistically significant improvement. Participants provided positive feedback on the intervention. A proportion of 59.9% believed the project was very useful, while 38.8% found it helpful. The qualitative component, too, revealed favourable responses.

### 5.1. Effectiveness of the Intervention

Weerasekara et al.’s [7] study on food- and nutrition-related knowledge, attitudes, and practices among reproductive-age women in marginalised areas in Sri Lanka showed that the audience has a low level of nutritional knowledge. Most women hold a positive attitude about nutritional knowledge, but report poor healthy diet practices. The present study reported positive knowledge, attitudes, social norms, and behaviour intentions. However, only the level of awareness/knowledge, attitudes, and practices showed significant improvement. Social norms, self-efficacy, and behaviour intentions did not change.

### 5.2. Effectiveness of Using Mobile Phones

Despite systematic reviews of mHealth in LMICs showing positive effects on maternal and child health service delivery and utilisation [31], several researchers recommend that there is a need for evidence for the effectiveness of both, i.e., for the health system strengthening as well as to promote the behaviour change in society. Our study highlights the second point by increasing dietary practices among the target group.

The results showed that regular text-based phone usage is 56% and smartphone usage is 43.7% among targeted rural women, which justifies using text-based interventions. Furthermore, based on the current findings, there is a potential for delivering future social media or app-based education messages.

Alam et al. [22] assessed the association between phone-based messaging services and practices regarding childbirth and the care of mothers and neonates in Bangladesh. They found that low-cost mobile phone messages can positively influence maternal and child healthcare behaviours, such as delayed timing of the first bath, in resource-poor settings. This suggests that further studies are needed to detect significant changes with an adequate sample size. In response, our study results show an increase in breastfeeding practices.

A study in Bangladesh by Khan et al. [20] indicated a potential to address gaps in nutrition service delivery through mobile phones. It is essential, however, to consider the readiness of the recipients to accept the technology during the design and delivery of the intervention. In the current study, participation in the intervention and the response rate of 72.3% (720/996) (Figure 2) reflect the participants’ readiness for such interventions.

As identified in the previous literature, the study faced infrastructural and technical limitations in implementing mHealth in low- and middle-income countries (LMICs), ranging from low network capacity and low access to mobile phones to specific technical barriers. mHealth also relies on the broader, costly infrastructure of health systems, technology, and industry to come together to add real, long-term value [32]. Further studies should be conducted to identify similar barriers for service providers and recipients.

A qualitative study in Nepal [25] found that although pregnant women and mothers have access to other phones in their families, they prefer to use their own phones, and phone sharing is uncommon. However, based on a comparison of outcomes between owners and non-owners of mobile phones, our study found that irrespective of the ownership of the phone, there was a significant increase in knowledge/awareness.

The current study found no difference based on the type of mobile phone, feature or smartphone, because the intervention mainly shared text-based messages. SMS messaging is a simple, cost-effective automated intervention that can reach all mobile phone owners. While the effect size is small, potential health benefits are worth achieving.

The current study showed that the increase in the knowledge/awareness among mothers might be due to the content of the messages and the follow-up calls and because mothers had not received regular services because of the COVID situation.

### 5.3. Importance of mHealth

The current study has confirmed the value of mHealth in promoting nutrition and improving maternal and child health. The project also developed local researchers’ capacity in nutrition education, early childhood development, and assessment in Sri Lanka. mHealth has evolved significantly over the last few years. Some programs in LMICs are incorporating this technology; however, governments have also not fully incorporated it into their primary healthcare systems [32]. As mHealth delivers behaviour change, service utilisation, and health outcomes at low cost, the LMICs should incorporate this technology in national policies and guidelines on nutrition, starting in a local area and scaling it up to the national level.

### 5.4. Value of a Theoretical Framework to Evaluate an Intervention

HOEM was used to evaluate the present study. The results provide evidence that the HOEM is a capable theoretical framework for evaluating the effectiveness of delivering messages using mobile phones and in lower middle-income settings in diverse cultural contexts. The results reveal the value of applying a theoretical framework in a community-based intervention and add to the evidence of applying theory and frameworks in message-sharing with mobile phones, social media, and mass media campaigns [29].

The HOEM’s applicability confirms a similar study to assess a campaign associated with physical activity-related beliefs, intentions, and physical activity [33] and to measure diverse health behaviour outcomes [34]. The current study also confirms that increased awareness and understanding may have led to behaviour change [35], although this relationship was not explicitly tested.

## 6. Limitations

There were several limitations of the study. The drop-out rate of 27.7% could be considered high in this study. However, it is compatible with the expected response rate of a telephone survey [36].

The validity of the responses given on the dietary history mainly depends on the accuracy of the responses given by the participants. Similarly, the practices mentioned in the results section were based on self-reported responses. Since there is no objective way of assessing the accuracy of those responses, the presence of social desirability bias cannot be excluded. Another limitation of this study is that most of the study sample comprises nursing women; however, it is unlikely that the responses of pregnant and nursing women would differ.

The utilisation of mass media channels would potentially influence the effectiveness of the interventions. For example, if a series of educational activities are telecast in the mass media during the intervention period, whether the true chance of the knowledge is due to the intervention or not could be evaluated. These messages are covered in antenatal health education sessions.

As mentioned earlier, there was no difference based on the type of mobile phone (standard or smartphone) because the intervention mainly shared text-based messages, not utilising the smartphone features of interactive apps. While the intervention included a picture and the same text message to the smartphone users, it did not enhance the effects of the campaign on smartphone users.

In a study in a Ugandan village, health team members used nutrition and health-related educational videos displayed on mobile phones during their family visits to analyse mothers’ reflections on the video education. The interviews with them and the focus group discussions with mothers demonstrated that they found the video education useful; many mothers reported improving their child feeding and hygiene practices [16]. Educational videos are a promising method to maintain and improve the motivation of voluntary community health workers and promote correct child feeding and hygiene practices. Future studies could include such features in mobile apps and make them sharable.

Finally, further scientific explorations are needed with multivariate analyses to explore the effects of confounding factors, as the current study only tested the intervention effect with bivariate statistics.

## 7. Conclusions and Recommendations

The quantitative and qualitative participant feedback revealed that the mobile-based intervention was successfully implemented among lower-middle-income pregnant and nursing mothers in Sri Lanka. With similar participants’ characteristics in the pre-assessment and post-assessment groups, we conclude that messages significantly improved the participants’ nutrition-related knowledge, nutrition-friendly attitudes, and favourable practices.

This method can be used on a larger scale to raise nutritional awareness in the community. In addition, these interventions could be alternative or complementary approaches in situations such as pandemic-related lockdowns where there is difficulty in delivering in-person behaviour change programs. Furthermore, if policymakers would like to add such a feature as a permanent addition to their existing health services, they would benefit from knowing the cost of this type of intervention.

## Figures and Tables

**Table 1:** Description of the pre- and post-test questionnaires with hierarchy of effects model variables.

No.	Concept	Number of Questions	Dichotomous Questions	Likert Scale (1–5)	Example of Questions
1	**Awareness/Knowledge** Seen or heard the messages Unprompted and prompted Recall specific messages Increase knowledge and understanding	15	Yes	-	What nutrient supplements does the clinic provide during pregnancy? (Multiple answers) 1. Vitamin C 2. Iron 3. Folic acid 4. Calcium 5. Cannot remember 6. Do not know
2	**Attitude** Level of agreement Changed attitudes	5	-	Yes	It is essential to intake nutrient supplements provided by the clinic or doctor during pregnancy. 1. Strongly disagree, 2. Disagree, 3. Neither agree nor disagree, 4. Agree, 5. Strongly agree
3	**Social norms** Level of agreement Changed social norms	5	-	Yes	Child feeding is a role of the mother, and the father does not want to involve in it 1. Strongly disagree, 2. Disagree, 3. Neither agree nor disagree, 4. Agree, 5. Strongly agree
4	**Self-efficacy** Influential Shared with others Confidence in being able to adapt	5	-	Yes	Are you confident that you can eat at least two servings of vegetables, one leafy vegetable and two servings of fruit a day? 1. Yes, always, 2. Yes, sometimes. 3. Don’t know, 4. Can’t answer now, 5. No
5	**Intention** Increase intention to practice the messages heard and seen	3	-	Yes	Do you intend to get your husband’s support for both child feeding and caring? 1. Yes, always, 2. Yes, sometimes. 3. Don’t know, 4. Cannot answer now, 5. No
7	**Practices/Behaviour trial** Change short-term practice behaviours Long-term behaviour change	2	Yes		Minimum dietary diversity-related set of questions

**Table 2:** Description of the intervention themes and messages.

Theme	Messages
**Pregnancy Care**	Message 1: Attend the antenatal clinic regularly for the safety of the pregnant mother and the child. Follow medical advice.
	Message 2: Folic acid, iron, vitamin C, and calcium are essential for mother and child nutrition. Get these as prescribed.
	Message 3: During pregnancy, it is essential to keep your mind relaxed and physically. Get at least 7–8 h of sound sleep at a stretch.
**Breastfeeding, Complementary Feeding**	Message 4: Even if a nursing mother is infected with or suspected of having COVID, she should continue breastfeeding by following safety measures.A lactating mother can continue to breastfeed continuously even after receiving the vaccine for COVID-19.
	Message 5: The first hour after birth, start breastfeeding the baby. Give the baby only breast milk for the first six months. Breastfeeding according to the baby’s needs. Not on a schedule.
	Message 6: Let us immediately start with healthy complementary foods for the six-month-old baby.Breastfeed for the appropriate number of times for age two years and beyond.Give the children diversified food with high nutrition quality and adequate quantity from the beginning.
	Message 7: Your child to become smart, healthy, and active; when feeding, consider age-appropriate quality, quality, variety, and the number of meals.
**Diet and food**	Message 8: Include a variety of foods in your daily diet in proper amounts for a healthy life.
	Message 9: Add pulses and fish or eggs or lean meat to your diet.
	Message 10: Get used to eating fresh vegetables and fruits with different colours, flavours, and aromas.It prevents and controls diseases and also increases appetite.
	Message 11: Eat at least two vegetables, one green leafy vegetable and two fruits a day.
	Message 12: Less than a teaspoon (5 g) of salt per day is enough for a person. Limit high-salt intake. Minimise adding salt to food.
	Message 13: Limit the consumption of sweets, biscuits, sugar, and sugar substitutes.
**Gender-related**	Message 14: Always maintain a good and happy family environment. Avoid fights and domestic violence.
	Message 15: Avoid alcohol, cigarette smoke, tobacco, and drugs, as they are bad for your health. Always maintain your family harmony.
	Message 16: Let us always give the father’s love and care to the well-being of the mother and children. The father’s support is also critical in feeding the children.
	Message 17: It is the responsibility of the husband and family to take care of her with love during pregnancy and breastfeeding. Her physical and mental health helps the child to grow well.
**Cash Management**	Message 18: Focus on family money management. Avoid unnecessary expenses.Doing so can prioritise the health, nutrition, food security, education, and well-being of the entire family.
	Message 19: Think again if everything you buy is essential. Do not waste money on non-essentials. Meet the needs of all families with proper money management.

**Table 3:** Socio-demographic characteristics of the pre- and post-study samples.

Variable	Pre (*n* = 996)		Post (*n* = 720)	
	**Number**	**Percentage**	**Number**	**Percentage**
**District**				
Batticaloa	238	23.9	172	23.9
Mannar	162	16.3	113	15.7
Mullaitivu	100	10.0	80	11.1
Kalutara	113	11.3	80	11.1
Moneragala	207	20.8	159	22.1
Matale	176	17.7	116	16.1
**Respondent Status**				
Pregnant mother	157	15.8	120	16.6
1^st^ trimester	1	0.6	1	0.8
2^nd^ trimester	5	3.2	4	3.3
3^rd^ trimester	151	96.2	115	95.8
Nursing mother	839	84.2	600	83.3
Age *				
16–20 years	80	8.0	52	7.2
21–30 years	594	59.6	435	60.4
31–40 years	287	28.8	211	29.3
More than 40 years	17	1.7	12	1.7
**Language Reading**				
Sinhala	479	48.1	346	48.1
Tamil	516	51.8	374	51.9
English	1	0.1	0	0.0
Level of Education **				
No schooling and schooled up to Grade 11	751	75.4	529	73.5
Grade 13 and above	244	24.5	190	26.4
Mode of receiving the nutrition and gender-related messages ***				
Public Health Midwife	954	95.8	690	95.8
Friends	136	13.7	100	13.9
Newspapers	84	8.4	62	8.6
Television	163	16.4	123	17.1
Radio	31	3.1	28	3.9
Community-level awareness programs	139	14.0	106	14.7
Mobile phones—Text messages	7	0.7	4	0.6
Mobile phones—WhatsApp, Viber messages	11	1.1	9	1.3
Internet/Web/Facebook/YouTube	73	7.3	54	7.5
Posters/books/magazines	66	6.6	49	6.8
Leaflets	50	5.0	38	5.3
Medical Officer of Health (MOH) or additional MOH	189	19.0	145	20.1
**Ownership of the mobile phone ^#^**				
Owned personal phone	673	67.6	498	69.2
Husband’s	258	25.9	189	26.3
Any other family member in the house	35	3.5	18	2.5
Any other person in the house	27	2.7	13	1.8
A family member or any other person not in the same house	0	0.0	0	0.0
**Type of mobile phone ^#^**				
Feature phone, not a smartphone	558	56.0	382	53.1
Smartphone	435	43.7	336	46.7
**Usage of TV/Radio ^##^**				
Not exposed to TV or radio	122	12.2	84	11.7
Exposed to either TV or radio	524	52.6	392	54.4
Exposed to both TV and radio	346	34.7	240	33.3
**Social Media usage ^**				
Not exposed to any social media channels	495	49.7	323	44.9
Exposed to one social media channel	124	12.4	98	13.6
Exposed to two social media channels	189	19.0	152	21.1
Exposed to three social media channels	141	14.2	110	15.3
Exposed to four social media channels	47	4.7	37	5.1

(* Missing: (pre-18, post-10), ** missing: (pre-01, post-01), *** missing: (pre-05, post-05) ^#^ missing: (pre-03, post-2), ^##^ missing:(pre-04, post-04); ^ social media (WhatsApp/Viber/Facebook/YouTube).

**Table 4:** Awareness/knowledge, attitudes, social norms, self-efficacy, and behaviour intentions of the entire sample.

Variable	Pre-Test Mean (SD)	Post-Test Mean (SD)	t-Value
Awareness/knowledge	3.78 (1.13)	4.86 (1.14)	−18.70 **
Attitude	15.21 (2.40)	15.42 (2.30)	−2.00 *
Social norms	7.88 (1.43)	7.87 (1.67)	0.14
Self-efficacy	4.29 (0.87)	4.36 (0.97)	−1.35
Behaviour intention	5.17 (0.77)	5.23 (0.81)	−1.38

** *p* < 0.01, * *p* < 0.05.

**Table 5:** Awareness/knowledge, attitudes, social norms, self-efficacy, and behaviour intentions of the sample based on the type of mobile phone ownership and type.

Variable	Pre-Test Mean (SD)	Post-Test Mean (SD)	t-Value
Sample who owned any type of a personal mobile phone			
Awareness/knowledge	3.80 (1.17)	4.92 (1.15)	−15.56 **
Attitudes	15.00 (2.33)	15.36 (2.30)	−2.98 *
Social norms	7.79 (1.36)	7.81 (1.66)	−0.17
Self-efficacy	4.29 (0.87)	4.38 (0.96)	−1.53
Behaviour intention	5.19 (0.76)	5.24 (0.80)	−0.90
Sample who did not own a personal mobile phone			
Awareness/knowledge	3.72 (1.04)	4.71 (1.10)	−10.28 **
Attitudes	15.69 (2.49)	15.56 (2.48)	0.62
Social norms	8.09 (1.56)	8.00 (1.66)	0.56
Self-efficacy	4.31 (0.87)	4.33 (0.98)	−0.22
Behaviour intention	5.11 (0.78)	5.20 (0.81)	−1.11
Sample who owned a smartphone			
Awareness/knowledge	3.66 (1.11)	4.92 (1.15)	−15.06 **
Attitudes	15.33 (2.36)	15.57 (2.38)	−1.54
Social norms	7.84 (1.43)	7.91 (1.61)	−0.61
Self-efficacy	4.30 (0.85)	4.36 (0.95)	−0.75
Behaviour intention	5.14 (0.79)	5.22 (0.81)	−1.21
Sample who owned a feature phone			
Awareness/knowledge	3.89 (1.13)	4.80 (1.13)	−11.60 **
Attitudes	15.10 (2.43)	15.30 (2.33)	−1.31
Social norms	7.92 (1.43)	7.83 (1.71)	0.78
Self-efficacy	4.28 (0.89)	4.37 (0.99)	−1.20
Behaviour intention	5.19 (0.75)	5.23 (0.80)	−0.74

** *p* < 0.01, * *p* < 0.05.

**Table 6:** Practices of the entire sample (unless noted).

Variable	Pre-Test	Post-Test	t-Value
	**Mean (SD)**	**Mean (SD)**	
Breastfeeding practices	3.34 (0.64)	3.49 (0.64)	−5.65 **
Breastfeeding practices of those who owned any type of a personal mobile phone	3.34 (0.64)	3.48 (0.65)	−4.16 **
Breastfeeding practices of those who did not own a personal mobile phone	3.35 (0.64)	3.52 (0.62)	−3.86 **
Breastfeeding practices of those who owned a smartphone	3.31 (0.64)	3.45 (0.66)	−3.70 **
Breastfeeding practices of those who owned a feature phone	3.38 (0.64)	3.53 (0.62)	−4.20 **
	**Number (%)**	**Number (%)**	**Chi-square value**
Minimum Dietary Diversity (MDD-W): 24 h dietary diversity (10 food groups ^ϕ^)			
Percentage who consumed food groups of five or more than 5 (entire sample)	635 (91.6)	689 (96.9)	18.18 **
Percentage who consumed food groups of five or more than 5 (owned any type of a personal mobile phone)	441 (92.3)	479 (97.6)	14.15 **
Percentage who consumed food groups of five or more than 5 (who did not own a personal mobile phone)	192 (90.1)	207 (95.4)	4.43 *
Percentage who consumed food groups of five or more than 5 (who owned a smartphone)	290 (90.6)	323 (97.6)	14.33 **
Percentage who consumed food groups of five or more than 5 (who owned a feature phone)	343 (92.5)	363 (96.3)	5.19 *

(^ϕ^ 1. Grains, white roots and tubers, and plantains 2. Pulses (beans, peas, and lentils) 3. Nuts and seeds 4. Dairy 5. Meat, poultry, and fish 6. Eggs 7. Dark green leafy vegetables 8. Other vitamin A-rich fruits and vegetables 9. Other vegetables 10. Other fruits). ** *p* < 0.01, * *p* < 0.05.

## Data Availability

Not applicable.
